# Cardiac Resynchroniztion Therapy In Heart Failure: Recent Advances And New Insights

**Published:** 2003-07-01

**Authors:** V Bhatia, R Bhatia, S Dhindsa, A Virk

**Affiliations:** * Department of Medicine, Mercy Hospital of Buffalo, Buffalo, NY, 14220,USA; † State University of New York, Buffalo, NY, 14220,USA; ‡ University of Texas, Houston

## Abstract

Newer non-pharmacological therapies for heart failure are being evaluated for patients of congestive heart failure (CHF). Mechanical support with left ventricular assist devices and heart transplantation are reserved for the minority of patients who have severely decompensated heart failure. Despite these therapeutic advances, it is generally accepted that current therapies do not adequately address the clinical need of patients with heart failure, and additional strategies are being developed. Cardiac resynchronization therapy (CRT) is a new modality that involves synchronization of ventricular contraction and has shown a lot of promise in managing symptomatic patients of CHF who are on optimal medical therapy and have interventricular conduction delay (IVCD). It has improved exercise tolerance and NYHA functional class in such patients in sinus rhythm and a recent meta-analysis has also shown mortality benefits in CHF. Recently benefits of CRT have also been observed in CHF patients who do not have wide QRS complexes on electrocardiogram (EKG). It has also been shown to benefit drug refractory angina in CHF. Recent studies have also focused on the combined use of CRT and implantable cardioverter defibrillator (ICD) and it has shown encouraging results. Our aim in this descriptive review is to define practice guidelines and to improve clinicians' knowledge of the available published clinical evidence, concentrating on few randomized controlled trials.

## Introduction

Approximately 30 percent of patients with cardiomyopathy have IVCD such as left or right bundle-branch block, leading to loss of coordination of ventricular contraction [1,2]. This dyssynchronous pattern of ventricular contraction is believed to contribute to the pathophysiology of heart failure, reducing the already diminished contractile reserve of the heart [3]. Specifically, dyssynchronous contraction exacerbates inefficient use of energy by the heart (a process termed mechanoenergetic-uncoupling [4]). The finding of IVCD has been associated with clinical instability and an increased risk of death in patients with heart failure [5-8].

Accordingly, the idea that cardiac-pacing technology might be used to restore the synchrony of ventricular contraction has been of theoretical interest for over a decade. Pacing modalities that utilize biventricular (BiV) or left ventricular (LV) stimulation to optimize cardiac pump function through synchronization of ventricular contraction are referred to as resynchronization or ventricular resynchronization therapies [2]. Resynchronization therapies can be present in a single device, in a device equipped with bradycardia pacing support, or incorporated into an ICD [9].

## CHF and IVCD

The most common causes for an IVCD in patients with heart failure are delayed left ventricular activation and left bundle branch block (LBBB). Impaired left ventricular function is also seen in otherwise normal subjects with isolated LBBB [10]. Approximately 20 to 30 percent of patients with symptomatic heart failure have an IVCD [2]. In a study done by Farewell et al [11] patients with a hospital diagnosis of "heart failure" were investigated. These patients did not undergo cardiac catherterization. The criteria for inclusion were severe heart failure (NYHA Class III or IV), heart failure due to dilated cardiomyopathy, QRS duration > 120 ms, or the presence of LBBB or RBBB. Using these criteria, approximately 10 percent of an unselected group of heart failure patients who are admitted to a typical district general hospital in United Kingdom during a calendar year would be candidates for biventricular pacing. A recent study done by Erdogan et al [12] estimated that biventricular pacing might be considered as an adjunct to standard heart failure therapy in 5-10 patients per year per 100,000 residents in industrial countries.

 In Europe Resynchronization therapy is approved for symptomatic heart failure that occurs in the setting of IVCD or BBB. This approval was granted on the basis of several studies of acute resynchronization therapy and data compiled in approximately 150 patients receiving BiV or LV stimulation for three months as part of two controlled studies (InSync [13,14] and PATH-CHF [15]). In the United States, resynchronization therapy with or without an ICD is approved for patients with NYHA class III-IV heart failure on the basis of the chronic studies described below, which were all performed with a control group randomly assigned to no resynchronization therapy [16-22].

There is another setting in which resynchronization might be important. It is estimated that approximately 8 to 15 percent of patients with advanced heart failure have pacemakers implanted for symptomatic bradycardia. Such patients have an increased risk of mortality or urgent transplantation due to progressive pump dysfunction; in one series, the risk at one year was 49 versus 15 percent in patients without a pacemaker) [23]. This difference may be due in part to the dyssynchronous contraction caused by right ventricular (RV) based pacing.

Whether such patients would derive long-term benefit from "upgrading" these devices to resynchronization therapies by the addition of a LV lead is currently under investigation. Initial data in patients with severe heart failure, prior atrio-ventricular (AV) junction ablation for rate control of AF, and chronic RV pacing has shown that there are significant benefits by upgrading from RV to BiV pacing [24].

## Effect on Contractile Function

Hemodynamic data acquired in patients with heart failure and bundle branch block (BBB) during acute or chronic BiV or LV stimulation have consistently shown improvements in measures of contractile response, such as force of contraction, cardiac output, left ventricular ejection fraction (LVEF), and pulmonary artery pressure, when compared to normal sinus rhythm or RV pacing [25,32]. CRT has been shown to decrease the functional mitral regurgitation in advanced systolic heart failure [33,34]. In contrast to other therapies that increase myocardial contractility, BiV and LV stimulation appear to modestly reduce myocardial energy demands and myocardial oxygen consumption [3]. The magnitude of acute systolic improvement by CRT is mainly due to resynchronization rather than due to change in myocyte function. An increased mechanical efficiency without increase in oxygen demand can be effective in drug refractory angina in CHF. A study done by Gasparini et al [35] showed the beneficial effects of CRT, during a mean follow-up of 9 months, in increasing the angina threshold in severely symptomatic patients with CHF and coronary artery disease (CAD) not amenable to cardiac revascularization. This study suggested that CRT increases the ischemic threshold in CHF patients on the long term, by markedly reducing the incidence of drug refractory anginal episodes, and by increasing a previously profoundly reduced exercise capacity. In another series of 18 patients with dilated cardiomyopathy (DCM) and an IVCD, aortic and LV pressures, dp/dt, and pressure-volume measurements were obtained during stimulation at single RV endocardial sites, at single LV epicardial sites, or during BiV pacing [36]. There was an improvement in systolic pressures with LV free wall or BiV stimulation, primarily due to an improvement in systolic function; there was no benefit on diastolic filling pressure or relaxation and RV apical or septal stimulation did not produce any hemodynamic changes. The markers of sympathetic activation, such as serum norepinephrine and heart rate variability, often vary directly with the severity of heart failure, these markers have not predictably changed in patients in whom resynchronization therapy appears to improve contractile function [37-40]. The improvement in mechanical synchrony appears to be the mechanism for reverse remodeling [41].

## Reverse Remodeling

Based upon echocardiography, preliminary data from the MIRACLE trial [16] suggested that BiV pacing is associated with reverse remodeling in patients with heart failure. BiV pacing produced an improvement in cardiac structure and function with a significant reduction in mitral regurgitation jet area and left ventricular mass, both signs of reverse remodeling [42]. Reverse remodeling was also observed in the CONTAK CD, PATH-CHF, and VIGOR CHF trials, in which BiV produced a significant reduction in left ventricular end-systolic and end-diastolic dimensions on echocardiography [38,43]. In the PATH-CHF trial, baseline left ventricular end-diastolic volumes were significantly smaller in those who exhibited reverse remodeling with BiV pacing compared to those who did not have a reduction in left ventricular volume [43].

## Clinical Trials

There are a number of trials evaluating the role of resynchronization therapy in patients with heart failure due to systolic dysfunction. The usual inclusion criteria include symptomatic heart failure that is stable on medical therapy, New York Heart Association (NYHA) class II to IV, left ventricular ejection fraction (LVEF) <35 percent, QRS duration >120 to 140 ms, and, in some trials, an indication for an ICD.

## MIRACLE Trial

In this trial16 453 patients with moderate-to-severe symptoms of heart failure associated with an LVEF of 35 percent or less and a QRS interval of 130 ms or more were studied. They were randomly assigned to a cardiac-resynchronization group (228 patients) or to a control group (225 patients) for six months, while conventional therapy for heart failure was maintained. The primary end points were the NYHA functional class, quality of life, and the distance walked in six-minutes. As compared with the control group, patients assigned to cardiac resynchronization experienced an improvement in the distance walked in six-minutes (+39 vs. +10 m, P=0.005), functional class (P<0.001), quality of life (-18.0 vs. -9.0 points, P= 0.001), time on the treadmill during exercise testing (+81 vs. +19 sec, P=0.001), and ejection fraction (+4.6 percent vs. -0.2 percent, P<0.001). In addition, fewer patients in the group assigned to cardiac resynchronization than control patients required hospitalization (8 percent vs. 15 percent) or intravenous medications (7 percent vs. 15 percent) for the treatment of heart failure (P<0.05 for both comparisons). Implantation of the device was unsuccessful in 8 percent of patients and was complicated by refractory hypotension, bradycardia, or asystole in four patients (two of whom died) and by perforation of the coronary sinus requiring pericardiocentesis in two others.

## MUSTIC Trial

The MUSTIC (Multisite Stimulation in Cardiomyopathies) trial is a single-blind randomized, controlled crossover study involving 131 patients who were divided into two groups based upon their underlying rhythm [17-19]. Group one included 67 patients with NYHA class III heart failure, QRS duration >150 ms with stable sinus rhythm and no conventional indications for pacemaker therapy [17]. The patients were randomly assigned to BiV pacing or no BiV pacing for three months, after which the pacing modes were switched; a total of 48 patients completed both phases of the study (MUSTIC SR). Exercise tolerance, as measured by the six-minute walk distance, increased by 23 percent after BiV pacing (399 versus 326 m, p<0.001). Other significant improvements included a 32 percent increase in quality of life, an 8 percent increase in peak oxygen consumption, and a two-thirds reduction in hospitalizations. Furthermore, BiV pacing was preferred by 85 percent of patients. At the end of the six-month crossover phase, the patients were programmed to the phase they preferred or, if there was no preference, according to the physician's judgment; almost all patients ended up with BiV pacing. The benefits with BiV pacing compared to baseline were maintained at 12 months19. Group two included 59 patients with heart failure and chronic atrial fibrillation (AF) with a wide QRS complex that required a permanent pacemaker because of a slow ventricular rate (MUSTIC AF). These patients were randomly assigned to either single site RV pacing or BiV pacing in the same fashion as in group one [18,19]. Only 37 patients completed the six-month crossover trial, which limits any conclusions that can be drawn [17]. Using an intention-to-treat analysis, there were no significant differences in exercise tolerance or peak oxygen consumption. In contrast, when only the 37 patients who completed the study were evaluated, biventricular pacing was associated with a significant increase in six-minute walking distance (9.3 percent, 32 meters) and peak oxygen consumption (13 percent, 1.7 mL/kg per min). At the end of the six-month crossover phase, 33 of 37 patients (89 percent) preferred BiV pacing. Among 33 patients followed at one year, significant improvements persisted in both six-minute walking distance and peak oxygen consumption.

## CRT and Implantable Cardioverter Defibrillator (ICD) Therapy

Recently there has been some trials evaluating the combined use of CRT and ICD in patients of heart failure.

### I

InSync trial [44] demonstrated the efficacy and safety of implanting a combined device in 362 patients with class III and IV heart failure who also required an ICD. Patients were randomly assigned to have BiV pacing turned on or off; the ICD was active in all patients. InSync ICD Italian Registry [45] studied InSync ICD model 7272, a dual chamber ICD combined with CRT. In this registry, CRT combined with ICD implantation has been feasible with few device or left pacing lead related complications and this was found to be concordant with previous reports [9]. The clinical benefits match those obtained in recipients of biventricular pacemakers, both in LVEF and NYHA functional class [16].

### II

VENTAK CHF/CONTAK CD [22] enrolled 581 patients with heart failure, most of whom had an ischemic cardiomyopathy, who also had an indication for an ICD. The majority of patients were male, had NYHA class II to IV heart failure, and a QRS duration >120 ms. The study utilized an ICD system designed to provide BiV pacing. All patients had an ICD and either BiV pacing or no pacing, each for six months.

### III

The COMPANION trial [46] is a study of resynchronization therapy with and without an ICD in patients with NYHA class III-IV heart failure who had a hospitalization for heart failure within the year prior to enrollment. Nearly half of all patients enrolled had a non-ischemic etiology of heart failure. Patients were randomly assigned to optimal medical therapy, resynchronization alone, or resynchronization with an ICD. The trial was discontinued in November 2002 due to a significant benefit in the combined end point of total hospitalizations and mortality among the device treated patients.

## Effects Of CRT and ICD Therapy on Arrhythmic Burden

Preliminary reports suggest that BiV pacing has an anti-arrhythmic effect [43,47]. The anti-arrhythmic effect has been attributed to improved hemodynamics. A low mean number of ventricular arrhythmic episodes were observed in the whole population and in the patients without Class I indications in InSync Italian registry [45]. It was found that the patients without standard ICD indications sustained serious arrhythmic events, confirming their high risk of death.

## Pacing Sites

Short-term studies have suggested that the lateral wall is a preferred site of LV stimulation to achieve effective CRT [3,25]. However, this choice may be limited by technical difficulties like high capture threshold from the presence of scar or fibrosis, particularly in patients with CAD, determining high LV pacing threshold, unfavorable coronary venous anatomy with narrow and tortuous coronary sinus tributary (CST), phrenic nerve stimulation or pacing lead instability. Gasparini et al [48] conducted a study to evaluate the effects of different pacing sites in patients treated with CRT. The data from this study revealed that, during long term follow-up, the most important clinical and echocardiographic parameters improved significantly in the patients, independently of the stimulation site. This was the case when considering each CST separately, or when dividing patients between "lateral" and "septal" sites, in the entire population and in the subgroups of patients without CAD. Hence in the presence of major technical difficulties preventing stimulation of the lateral LV, alternative-pacing sites, particularly the basal anterior LV wall, may be suitable to offer effective CRT to these patients. Tissue Doppler echocardiography has also been used to determine the optimal pacing site for BiV pacing [47] and to document an improvement in LV function, manifest by an increase in LV and interventricular synchrony, a shortened isovolumic contraction time, and an increased diastolic filling time [41,50].

## QRS Duration And Dyssynchrony

Wide QRS duration, possibly with LBBB, has been proposed as an independent predictor of total mortality in CHF patients [51-53]. In addition it has been considered a key criteria for selecting CHF patients for CRT, as wide QRS has been suggested to be associated with marked RV to LV and intra-LV dyssynchrony [10,25,54]. However, the correlation between the QRS width and regional electromechanical LV dyssynchrony has not been completely clarified [47,55,56] and a high prevalence of left ventricular systolic and diastolic asynchrony has been found in patients with congestive heart failure and normal QRS duration [57]. Hence for a given QRS width there is a considerable scatter in response to CRT responsive patients with narrow complexes and less responsive ones with wide complexes exist [32]. A study was done by Gasparini et al [58] to assess in a large cohort of patients the role of baseline QRS width (<150 / ≥150 ms) on clinical and echocardiographic parameters, hospitalization rates, and survival after CRT. In this study 158 CHF patients (121 men, mean age 65 years, mean LVEF 0.29, mean QRS width 174 ms) underwent successful BiV implantation and were then followed for a mean time of 11.2 months. According to the basal QRS duration, patients were divided in two groups with wide QRS group (≥150 ms, 128 patients, 81 percent) and the narrow QRS (<10 ms, 30 patients, 19 percent).

 In the wide QRS group, following results were noted:

1. LVEF improved from 20 percent to 39 percent (P < 0.0001)
2. Six-minute walk test from 311 to 463 m (P<0.0001)
3. NYHA Class III-IV patients decreased from 86 percent to 8 percent (P<0.0001).

In the narrow QRS group, following were the results:

1. LVEF improved from 30 percent to 38 percent (P<0.0001).
2. Six-minute walk test 370 to 506 m (P<0.0001).
3. NYHA Class III-IV patients decreased from 60 percent to zero percent (P<0.0001).

The data showed that in wide and narrow QRS patients, BiV pacing significantly improved clinical parameters (NYHA lass, six-minute walk test, quality of life, and hospitalization rate) and main echocardiographic indicators. Furthermore, narrow QRS patients had a better survival rate, rapidly regained left ventricular function, and only a few patients remained in a higher NYHA class during follow-up. These patients should not be excluded "a priori" from CRT.

This study highlights the important point at what level a QRS has to be considered "wide enough" to be proposed to benefit from CRT. For example this was >150 ms for the MUSTIC study17, >130 ms in the MIRACLE study16, and >120 ms in Comparison of medical therapy, pacing, and defibrillation in chronic heart failure (COMPANION) trial [46]. It is evident from these differences in opinion that there is still no consensus on just how "wide" QRS should be for an efficacious CRT. Moreover the duration of QRS alone no longer seems to be defining parameter for patients with either inter-or-intraventricular dyssynchrony, given that recent studies have shown that even patients with a QRS<150 ms or without LBBB can suffer from significant dyssynchrony [53,54].

## Are There Any New Markers of Asynchrony?

The results of the recent investigations have prompted a reappraisal of the apparent correlation between conduction disorders and cardiac dyssynchronization. In a tissue Doppler study of 104 patients with BBB, Garrigue et al [56] observed that 35 percent of patients with LBBB had no interventricular dyssynchronization, and 20 percent had no left ventricular dyssynchronization. Despite fulfilling the "classic" criteria of wide QRS and LBBB, these patients are hardly candidates for CRT. Conversely, a sizable number of patients with right bundle branch block (RBBB) may present with mechanical anomalies, which may be corrected by CRT. Therefore, new markers of asynchrony are desirable, more directly related to cardiac mechanical function than the EKG. Among several methods available, angioscintigraphy with phase analysis of the contraction isochrones was the first, though its cumbersome implementation, high cost, and limited availability in routine clinical practice have prevented its widespread application [59,60].

A recent study done by Cazeau S et al [61] explored the value of an echocardiographic model to identify cardiac electromechanical dyssynchrony parameters (EDP) in candidates for CRT and their potential correction after implantation. The study included 66 CRT recipients of CRT NYHA functional class III or IV who had one or more AV, interventricular or intra-ventricular dyssynchrony criteria. An immediate improvement was observed in 85 percent of the population with partial or total correction of their EDP. However the modification in EDP differed considerably between recipients of de novo CRT systems and patients with previously implanted standard pacing systems upgraded with the implantation of a left ventricular lead. EDP measurements appear to identify candidates for CRT and to confirm the success of system implantation. This is the first report of a selection of candidates for CRT based on mechanical instead of electrical criteria. An overlap certainly exists between patients presenting with a wide QRS and patients with disorders of cardiac synchronization. However the echocardiographic method, which distinguishes three different types of synchronization offers a finer analysis of the anomalies amenable to resynchronization.

## Etiology of CHF and CRT

The mechanisms of CHF in patients with DCM are complex and multiple. A study done by Gasparini et al et al [61] examined the importance of underlying cardiac pathology on the outcome of CRT, hypothesizing that myocardial infarction scar and the non-contractile segment represent limitations to the ability to resynchronize cardiac contraction in patients with CHF associated with DCM. The results of this study showed that the functional capacity improved significantly during CRT in CAD and non-CAD patients. LVEF and NYHA class in non-CAD patience showed a significantly greater improvement. However, changes in quality-of-life were similar in both groups. The mechanisms of slow myocardial conduction associated with asynergic contraction in patients with DCM vary with the underlying pathology et al [5,62,63]. In patients with DCM not due to CAD, ventricular asynergy may be associated with interventricular or intra-ventricular conduction delays. Interventricular asynergy is most often associated with LBBB. A progressive remodeling of myocardial collagen matrix well documented in familial cardiomyopathies may impair intraventricular conduction. Disruption of collagen network, by altering the cellular architecture, impairs intraventricular conduction and the coordinated mechanical response of the ventricles. The consequences are QRS prolongation and waste of mechanical work. In patients with CAD, beside ventricular remodeling, ventricular asynergy may be associated with segmental wall- motion abnormalities as result of myocardial infarction scars, or of ischemic non-contractile segments. Segmental wall motion abnormalities affect intraventricular conduction and the coordinated mechanical response of the ventricles. CRT may correct conduction delay in remodeled dilated myocardial segments, but has no effect on extensive myocardial scars or ischemic segments. CRT can only recruit and coordinate a fraction of the myocardial mass to increase ventricular mechanical work in patients with CAD. Although significant benefits were observed in both groups after CRT, myocardial infarction scars limit the mechanical benefits of QRS narrowing and resynchronization. Hence the benefits of CRT should not be denied to patients with severe CHF on the basis of underlying cardiac pathology including patients with severe LV dysfunction associated with CAD and wide QRS et al [64].

## Pacing in AF

Paroxysmal or persistent AF occurs in up to 30 percent of patients with HF [65]. Rate control can be achieved with pharmacological therapy. In patients refractory to such therapies these objectives can be achieved with radiofrequency ablation of the AV node and pacemaker therapy with traditional RV-based pacemakers. Initial data regarding "upgrading" from RV to BiV pacing using an LV lead to achieve cardiac resynchronization in heart failure patients with chronic AF who have undergone radiofrequency AV nodal ablation followed by standard RV pacing is promising [24].

## Recommendations

BiV pacing is an effective approach to the therapy of patients with heart failure and IVCD and studies suggest that BiV pacing can improve exercise tolerance and NYHA functional class in such patients in sinus rhythm. A meta-analysis was done by Bradley et al [66] of the available studies to determine the effect of CRT on mortality in CHF. 11 reports of 4 randomized trials with 1634 total patients were included in this meta-analysis. It was found that cardiac resynchronization reduces mortality from progressive heart failure in patients with symptomatic left ventricular dysfunction. This finding suggests that cardiac resynchronization may have a substantial impact on the most common mechanism of death among patients with advanced heart failure. Cardiac resynchronization also reduces heart failure hospitalization and shows a trend toward reducing all-cause mortality.

More data on effect of CRT on mortality in CHF is still awaited. Although suggestive, the data are insufficient to prove efficacy in patients in AF [18]. As a result of the MIRACLE trial, the FDA has approved BiV pacing as a treatment for moderate to severe heart failure. Potential concerns include the small risk of serious complications during implantation as noted in MIRACLE [16] and lack of data concerning the long-term effects of cardiac resynchronization as noted by the 2001 Task Force of the ACC/AHA [67].

At present, it seems reasonable to consider BiV pacing in patients with a low LVEF and prolonged QRS duration who remain symptomatic (NYHA class III or IV HF) despite optimal medical therapy [68]. The 2002 task force of the ACC/AHA/NASPE gave a class IIa recommendation (weight of evidence in favor of efficacy) to BiV pacing in medically refractory, symptomatic NYHA class III or IV patients with idiopathic dilated or ischemic cardiomyopathy, prolonged QRS interval (130 ms), LV end-diastolic diameter greater than or equal to 55 mm and ejection fraction less than or equal to 30 percent.

It is not known if these devices should routinely incorporate a defibrillator. The MADIT II trial showed a significant survival benefit from ICD placement in patients who have had a previous myocardial infarction and have an LVEF 30 percent [69]. Combination therapy with an ICD and BiV might therefore be beneficial in such patients who have QRS prolongation.
